# Modeled versus Experimental Salt Mixture Behavior under Variable Humidity

**DOI:** 10.1021/acsomega.4c01486

**Published:** 2024-03-27

**Authors:** Sebastiaan Godts, Michael Steiger, Amelie Stahlbuhk, Scott Allan Orr, Julie Desarnaud, Hilde De Clercq, Veerle Cnudde, Tim De Kock

**Affiliations:** † Monuments Lab, Royal Institute for Cultural Heritage (KIK-IRPA), Brussels 1000, Belgium; ‡ Antwerp Cultural Heritage Sciences (ARCHES), University of Antwerp, Ghent 9000, Belgium; § Department of Geology (PProGRess), Ghent University, Ghent 9000, Belgium; ∥ Department of Chemistry, University of Hamburg, Hamburg 20146, Germany; ⊥ Institute for Sustainable Heritage, University College London (UCL), London WC1E 6BT, United Kingdom; # Department of Earth Sciences, Utrecht University, Utrecht3584 CS, The Netherlands

## Abstract

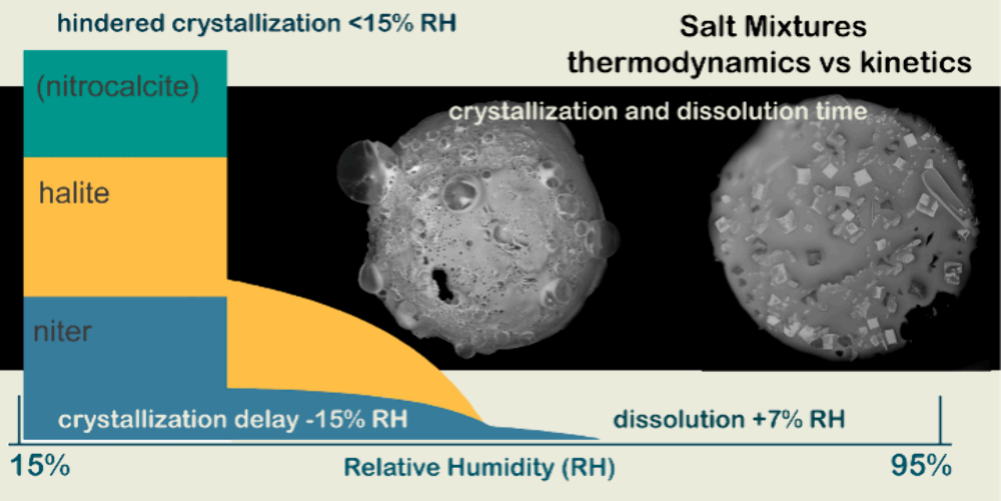

This study investigates the kinetics of salt mixture crystallization under relative humidity (RH) conditions, varying between 15 and 95% (at 20 °C), to inform applications in built heritage preservation, geology, and environmental sciences. We focused on commonly found, sulfate-rich and calcium-rich salt mixtures containing five to six ions, Cl^–^, NO_3_^–^, Na^+^, and K^+^, including or excluding less common Mg^2+^, and including either an excess of SO_4_^2–^ or Ca^2+^, with respect to gypsum. Using time-lapse micrographs and dynamic vapor sorption, we explore how crystallization and dissolution behavior depend on RH and mixture composition under constant temperature. A range of RH change rates were studied to simulate realistic weather events. Microstructural analyses through environmental scanning electron microscopy (ESEM) confirmed the crystal habit corresponding with RH transitions. Phases predicted from thermodynamic modeling (ECOS/RUNSALT) were confirmed using micro-Raman spectroscopy, X-ray diffraction (XRD), and elemental mapping via energy-dispersive X-ray spectroscopy (EDX). We identify a strong correlation between phase transition kinetics and RH change rates, with crystallization deviating by −15% and dissolution by +7% from modeled values under rapid (several seconds) and slow (several days) RH changes. These insights are important for preservation strategies in built heritage, salt deposition, and dissolution mechanisms in diverse geological and realistic environmental contexts, laboratory experiments, future modeling efforts, and the understanding of stone decay in general.

## Introduction

Built heritage faces challenges that are extensive in a rapidly changing society and climate. Salts and consequential weathering are considered understudied and vital to establishing proper conservation management strategies. Salt crystallization–dissolution cycles have the potential to weaken and break down porous materials, which ultimately leads to loss of the integrity, function, and value of cultural heritage. However, salt behavior is a complex subject due to the presence of a wide variety of ions, which are often the result of groundwater infiltration by capillary rising dampness, rainwater infiltration, and atmospheric, biological, or internal material contamination. A wide range of literature is available considering the effects of salt crystallization in porous media, broadly defined as salt weathering with several important publications on the impact on natural stone materials such as the milestone references on natural landscape formation by Goudie and Viles^1^ and the review by Evans.^2^ In contrast, the literature about stone in the built environment has focused on practical approaches to addressing stone conservation (e.g.,^3−6^). Fundamental questions, such as the mechanisms of crystallization and the development of crystallization pressure in porous media, remain open questions and active areas of research (e.g.,^7−17^). Nevertheless, there is consensus that the occurrence of repeated cycles of crystallization and dissolution of hygroscopic salts is largely governed by changing conditions in relative humidity and temperature and that repeated crystallization leads to the degradation of porous stone materials through the resulting weakening of intergranular bounds in the substrate. Additionally, moisture stains and biological contamination can be problematic due to the hygroscopic nature of certain mixture compositions, even in the absence of liquid water.

Thermodynamic calculations are often used to understand the interactions of salts with the environment.^18^ However, few studies investigate the behavior of mixtures in the built environment subjected to different environmental conditions.^19−21^ Experimental verification of model outputs is important to increase confidence and identify issues related to the behavior of modeled salt mixtures.^22^ This includes the assessment of salt crystallization kinetics that are not considered in multi-ion models, thus allowing the interpretation of results to identify realistic phase transitions when advising preventive measures to mitigate and understand salt mixture behavior. The research presented focuses on common mixture compositions derived from a statistical analysis of 11,412 samples taken in 338 historic buildings (monuments and sites) primarily in Belgium.^23,24^ The mixtures of interest include seven ions of chloride (Cl^–^), nitrate (NO_3_^–^), sulfate (SO_4_^2–^), sodium (Na^+^), potassium (K^+^), magnesium (Mg^2+^), and calcium (Ca^2+^) and the possible solids that can crystallize. Given that gypsum, the salt with the lowest solubility in this system, remains in a crystalline form, its ions will not influence the solution’s properties. As a result, the remaining solution will contain either calcium or sulfate ions, depending on the composition of the mixture. Under these circumstances, the remaining ions are either Cl^–^, NO_3_^–^, SO_4_^2–^, Na^+^, K^+^, Mg^2+^ (type 1, sulfate-rich) or Cl^–^, NO_3_^–^, Na^+^, K^+^, Ca^2+^, Mg^2+^ (type 2, calcium-rich).^23^

Type 1 mixtures generally exhibit lower hygroscopic properties, tend to crystallize at relative humidity above 60%, and often include hydrated and double salts. In contrast, type 2 mixtures are more inclined to crystallize below 60% and frequently contain hygroscopic salts that are often subject to kinetic hindrances, delaying crystallization. In the built environment, 14 solids are frequently identified from mixtures. Here, we present experimental results on four salt mixtures (two of each type) with either five or six ions. A total of 11 solids are being examined, each appearing in at least 30% of a representative selection of mixtures commonly found in the built environment.^25^ In this study, we aimed to verify the modeled behavior of salt mixtures through droplet experiments. The approach includes identifying kinetic deviations from model predictions and exploring how a combination of techniques can enhance our understanding of crystallization and dissolution processes in general. High-resolution time-lapse micrographs, similar to experiments described by Desarnaud and Shahidzadeh-Bonn,^26^ are used to identify these processes. In this study, we investigate the behavior of mixtures instead of single salts through a windowed climate chamber and via dynamic vapor sorption to identify processes under rapid and slow rates of RH change at constant temperature, comparable to those in realistic environments. Solid phases and crystal habit are investigated with Raman spectroscopy, XRD, and ESEM-EDX.

## Methods and Materials

### Modeled Crystallization Behavior

The ECOS/RUNSALT^27,28^ model is used to calculate the crystallization behavior of four different mixture compositions (Table 1). Two different compositions are selected per mixture type based on their frequency of occurrence, as described in^25,29^. To recap, the ECOS model is based on the Pitzer–Simonson–Clegg model,^18^ including ion concentrations expressed as mole fractions. The outputs of the model are investigated to determine the crystallization behavior of salt mixtures under changing RH between 15 and 95% at 20 °C. The results are compiled from several outputs of the model, stitching together 5% RH ranges to achieve 0.1% RH resolution. Further input details, terminology, limitations, issues, and solutions for the model are taken into consideration, as described in^22^.

**Table 1:** Initial Mixture Composition (mol·kg^–1^)^a^

	**Cl**^**–**^	**NO**_**3**_^**–**^	**SO**_**4**_^**2–**^	**Na**^**+**^	**K**^**+**^	**Mg**^**2+**^	**Ca**^**2+**^
**mix T1**_**V**_	1.0	1.0	1.0	2.0	2.0	0.0	0.0
**mix T1**_**VI**_	1.0	2.0	1.0	2.0	1.0	1.0	0.0
**mix T2**_**V**_	1.9	4.7	0.0	1.9	1.9	0.0	1.4
**mix T2**_**VI**_	2.2	3.8	0.0	1.1	1.1	1.1	0.8

a Each mixture is given a sample name corresponding to either a type 1 mixture (sulfate-rich) = T1, or a type 2 mixture (calcium-rich) = T2, while subscripts _V_ and _VI_ refer to five or six ions.

The model outputs show solids that can crystallize from the solution. For example, at a given RH, the amount of crystalline solids is indicated. Because a limited number of independent variations of coexisting phases in a system are possible, generally known as the phase rule, a maximum of four and five solids can coexist at a given temperature (*T*) and relative humidity (RH) within the five- and six-ion mixtures. The salts under investigation in this study were

- aphthitalite (Na_2_SO_4_·3K_2_SO_4_)
- thenardite (NaSO_4_)
- mirabilite (NaSO_4_·10H_2_O)
- darapskite (NaNO_3_·Na_2_SO_4_·H_2_O)
- nitratine (NaNO_3_)
- halite (NaCl)
- niter (KNO_3_)
- bloedite (Na_2_SO_4_·MgSO_4_·4H_2_O)
- magnesium sulfate hydrates (MgSO_4_·*x*H_2_O)
- nitromagnesite (Mg(NO_3_)_2_·6H_2_O)
- carnallite (KCl·MgCl_2_·6H_2_O)
- sylvite (KCl)
- bischofite (MgCl_2_·6H_2_O)
- hydrated calcium nitrate (Ca(NO_3_)_2_·*x*H_2_O).

This analysis excludes the double salts Ca(NO_3_)_2_·KNO_3_·3H_2_O^30^ and Ca_2_Cl_2_·Ca(NO_3_)_2_·4H_2_O,^31^ as these solids are currently not considered in the model.

### Experimental Mixture Composition

The experiments were carried out with the solutions presented in Table 1, further defined as mixtures T1_V_, T1_VI_, T2_V_, and T2_VI_. Solutions were prepared with analytical grade salts (Merck KGaA, EMSURE) below the saturation degree to allow complete dissolution; the mixtures are considered to be saturated with respect to aphthitalite (T1_V_), epsomite (T1_VI_), and niter (T2_V_ and T2_VI_) at 20 °C.

### Time-Lapse Micrographs under Rapid-Changing RH

Dissolution and crystallization times were captured using time-lapse micrographs from a 3D-digital microscope (HIROX) with the following settings: 100× to 200× magnification, lens MXG-2500REZ, KH-8700, a diameter of 2079.49 μm field of view, and 1.30 μm spatial resolution. It is important to note that while the resolution of the micrographs impacts the granularity of the obtained data, the research prioritizes understanding the overall crystallization timeline over the exact moment of nucleation, due to the study’s focus on the decay of porous materials. The need for pore filling in such decay processes makes the specific timing of nucleation less important. Instead, the study concentrates on the delay and duration of complete crystallization, offering insights into the decay processes of porous materials, where the completion time of crystallization is more relevant to assessing the environmental impact. The processes were monitored in a windowed climate chamber with a 0.2 L/min constant gas flow of nitrogen with controlled RH (GenRH/Mcell, with a rotronic HC2-IC 102 high-temperature industrial humidity probe, accuracy: ±0.8% RH). Micrograph intervals of 2, 5, 30, or 60 s were chosen based on observed phase transitions in initial runs. All tests were conducted at a lab temperature of 20 °C (±1) and 15 to 95% RH. Solid phases were examined using a portable Raman spectrometer (Renishaw, Virsa) at specific RH levels where crystals became visible. After 3 months of conditioning at 15% RH and 20 °C, micro-Raman spectroscopy (Renishaw InVia) was performed for further verification.

**Figure 1:**
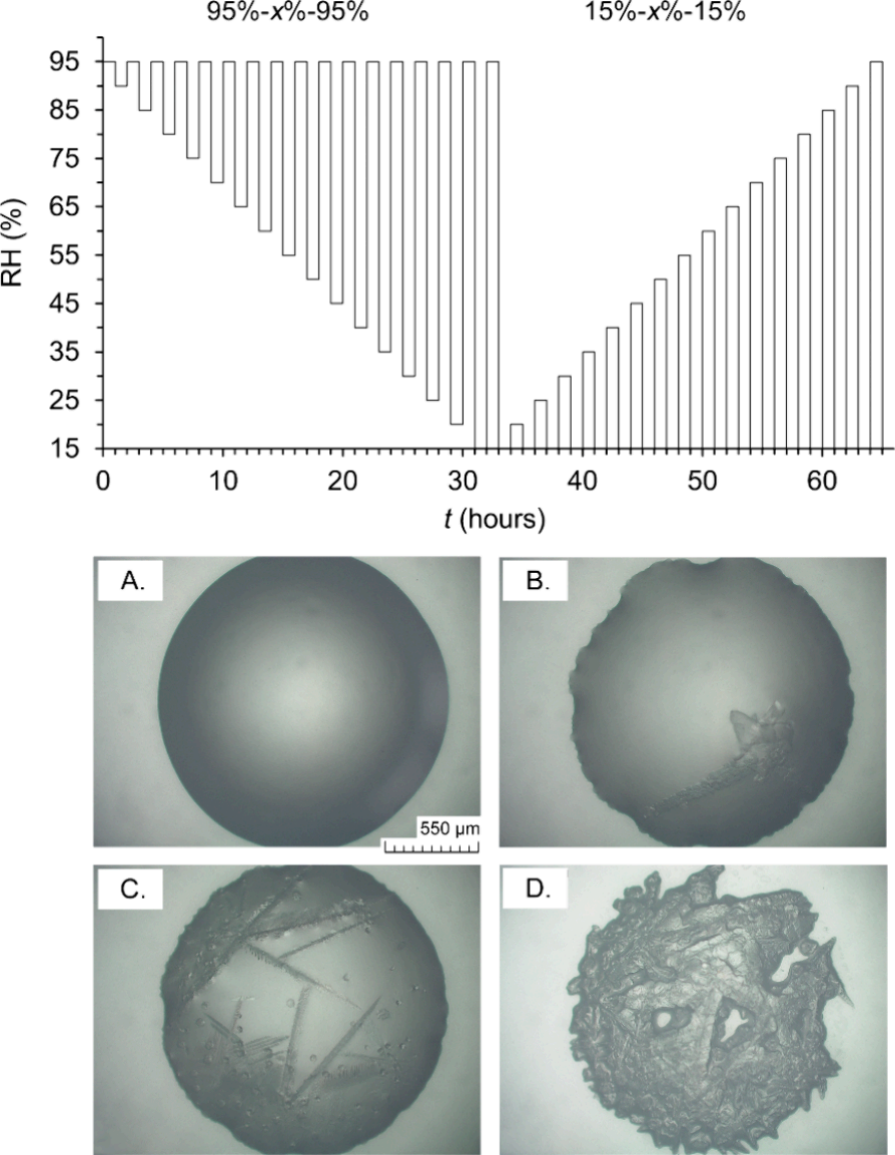
Top: Relative humidity cycles were used to explore crystallization and dissolution of salt mixtures (T1_V_, T1_VI_, T2_V_, T2_VI_) using time-lapse micrographs in a climate chamber. Bottom: Images A–D illustrate micrographs (0.5 μL initial volume) of a T2_VI_ droplet at 95% RH and its crystallization, respectively, at 30, 20, and 15% RH, at 20 °C. The micrographs quantify visible crystallization and dissolution times at different RH levels.

For both methods, Raman spectra were obtained using a 785 nm, 100–400 mW near-infrared diode laser and a long-distance objective at magnifications of 5×, 20×, or 50×. A 10 s exposure time and 100–2000 cm^–1^ measurement range were sufficient for identifiable spectra against an in-house reference library (refer to the Supporting Information). Lastly, X-ray diffraction (XRD) analysis (Bruker D8 in theta/2theta configuration) was performed on the dried samples after the same 3-month conditioning period. In each experiment, six 0.5 μL droplets of solution (per mixture: T1_V_, T1_VI_, T2_V_, and T2_VI_) were placed on an 18 mm × 18 mm glass slide within the windowed climate chamber. The droplets were initially conditioned at 95% RH and then dried at 15% RH, with each step lasting 1 h. Following this preconditioning, the droplets were subjected to RH cycles returning to either 95 or 15% RH after each intermediate step *x* (1 h) (Figure 1).

The mean rate of RH change is derived from the calculated slopes of the obtained data points. For rapid RH changes, the mean slope is approximately 0.6% RH s^–1^. A full procedure of one mixture includes up to 24,000 micrographs to identify the exact moment when visible crystallization occurs, how long the process takes, and vice versa the time for completed dissolution at a given RH. Specifically, the experimental procedure is as follows:

- For the cycles starting at 95%, the pattern is 95%–*x*%–95%, where *x* decreases from 90 to 15% RH in 5% steps. *x* = 90, 85, 80, 75, 70, and so forth until 15%.
- For the cycles starting at 15%, the pattern is 15%–*x*%–15%, where *x* increases from 20 to 95% RH in 5% steps. *x* = 20, 25, 30, 35, 40, and so forth until 95%.

The experimental series reaches a total of 66 steps: thus, a duration of 66 h per mixture type. RH and T were logged every 2 s in the climate chamber near the droplets. The experimental method involves identifying kinetic properties and deviations concerning the crystallization or dissolution RH and times (see below), which are correlated to the mutual crystallization or dissolution relative humidity for each solid as calculated (ECOS/RUNSALT).

### Vapor Sorption under Slowly Changing RH

Sorption and desorption isotherms of the four mixtures were determined via dynamic vapor sorption (SPSx-1 μ high load, ProUmid, SPS: sorption testing system, 1 μg resolution). All isotherms were recorded at 20 °C between 15 and 95% RH for sorption and desorption. For each run, 20 μL droplets of the mixed solution were placed in an aluminum sample pan of the SPS autosampler. To ensure reliability, data from four runs per mixture type were averaged to obtain mean values and standard deviations were calculated to assess the variability among replicates. The sample mass was recorded at 15 min intervals. Equilibrium conditions are met by performing a linear regression on net weights observed over the time period. The equilibrium gradient, determined by the slope of the regression line, is considered achieved if it falls within the specified limit, defined as change in mass of less than 0.01% per 40 min. The initial conditions were set to 40 °C and 15% RH to ensure a stable mass, reaching a mean equilibrium time for all mixtures of 2.8 h (standard deviation (SD) = 0.01 h), followed by the experiments carried out at 20 °C and RH steps of 2% each maintained for a maximum of 6 h or until equilibrium conditions were met. The mean experimental time of the latter was 159 h (SD = 5 h) for the sorption phase (15 to 95% RH) and 171 h (SD = 3 h) for desorption (95 to 15% RH), reaching a total experimental time of 330 h (SD = 8 h). Thus, the mean rate of change is approximately 0.5% h^–1^. Besides a general investigation of the hysteresis between sorption and desorption curves, the first derivative of the individual curves is calculated to identify RH points of interest where crystallization and dissolution occur.

Raman spectra and imaging were conducted separately during the experiments, with each method being performed in two separate runs out of the four total. Images were obtained at each RH step with a 50 mm lens and CMOS sensor (11.3 mm × 11.3 mm, 2046 × 2046 pixel resolution, 5.5 × 5.5 μm pixel size). A Wasatch Photonics WP 785 (nm laser) was used to obtain Raman spectra at approximately each 5% RH step, with the following parameters: laser power 450 mW (100% intensity), wavelength resolution 7 cm^–1^, 200 ms integration time, 2 scan average, 1 pixel boxcar smoothing, 270–2000 cm^–1^ spectral range, working distance 50 mm.

An additional experimental run was conducted, which included imaging, under slower conditions compared with previous runs. The primary difference in this run was that it was performed with two samples for each mixture type. RH steps of 2% were maintained for a maximum of 50 h or until equilibrium conditions were met. On average, the sorption phase (from 5 to 93% RH) took 885 h, while the desorption phase (from 91 to 5% RH) took 789 h. The total experimental duration amounted to 1674 h. Consequently, the average rate of change in relative humidity was approximately 0.1% h^–1^.

### Time Steps Considered to Identify Processes

**Figure 2:**
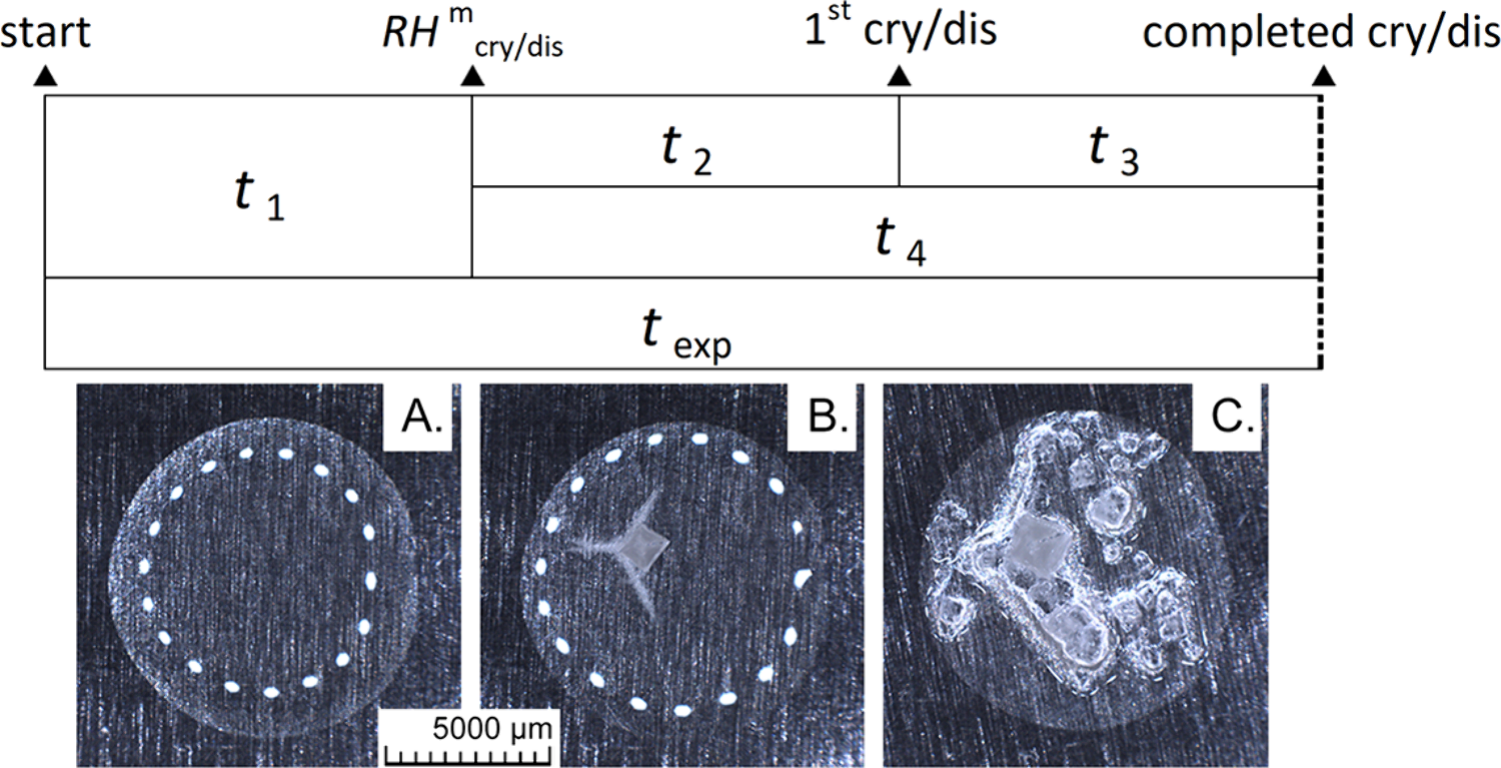
The table on the top illustrates the time intervals of the experiments. *t*_1_: from start of the RH step to first mutual (m) crystallization (cry) or dissolution (dis) RH target (as defined by the modeled behavior, ECOS/RUNSALT), *t*_2_: from the end of *t*_1_ until first visible crystal or dissolution (first cry/dis), *t*_3_: from the end of *t*_2_ until complete visible crystallization or dissolution (completed cry/dis); *t*_4_: *t*_2_ + *t*_3_, *t*_exp_: start of *t*_1_ until the end of *t*_3_, thus the total experimental time for each RH step. Panels A–C (bottom) display images acquired throughout the SPS experiments (initial volume 20 μL), illustrating a salt solution droplet (T2_V_) at 63% RH, initial crystallized salts at 49% RH, and completed crystallization at 15% RH and 20 °C, respectively. The white dots in panels A and B are the result of light reflection.

The effective crystallization and dissolution times observed by microscopy were recorded under rapid and slowly changing RH. As illustrated in Figure 2, we consider:

- *t*_1_ = the start time of the experiment until the first mutual crystallization or dissolution RH is reached, as modeled by ECOS/RUNSALT. Thus, approximately 0.6% s^–1^ for rapid (GenRH) and 0.5% and 0.1 h^–1^ for slow RH changes (SPS).
- *t*_2_ = from the end of *t*_1_ until the first visible crystal or dissolution, which is considered as the induction time.
- *t*_3_ = the time from the end of *t*_2_ until complete visible crystallization or dissolution, hence the effective crystallization/dissolution time.
- *t*_4_ = from the end of *t*_1_ to the end of *t*_3_, the induction time plus completed crystallization/dissolution, thus equal to *t*_2_ + *t*_3_.
- *t*_exp_ = the total experimental time from the start of *t*_1_ until complete visible crystallization or dissolution (end of *t*_3_). This can be less or exceed the experimental cycle time (1 h for rapid and maximum 6 and 50 h for slow changing RH)

### Investigating Crystal Habit

Environmental scanning electron microscopy combined with energy-dispersive X-ray spectroscopy (ESEM-EDX) was performed for all four mixtures (EVO system from Carl Zeiss Microscopy GmbH). The method aims to compare the crystal habit and elemental variations in salt mixtures after undergoing both rapid and slow (evaporation) crystallization. To ensure stable imaging and video capture at higher RH levels, the Peltier stage temperature was set to 5 °C, allowing higher vacuum. Key experimental parameters included an accelerating voltage (Extra High Tension, EHT) at 20.00 kV, a LaB6 (lanthanum hexaboride) cathode filament, and the use of an NTS BSD detector (nanoTechnology Systems BackScattered).

## Results and Discussion

### Modeled Crystallization Behavior

**Figure 3:**
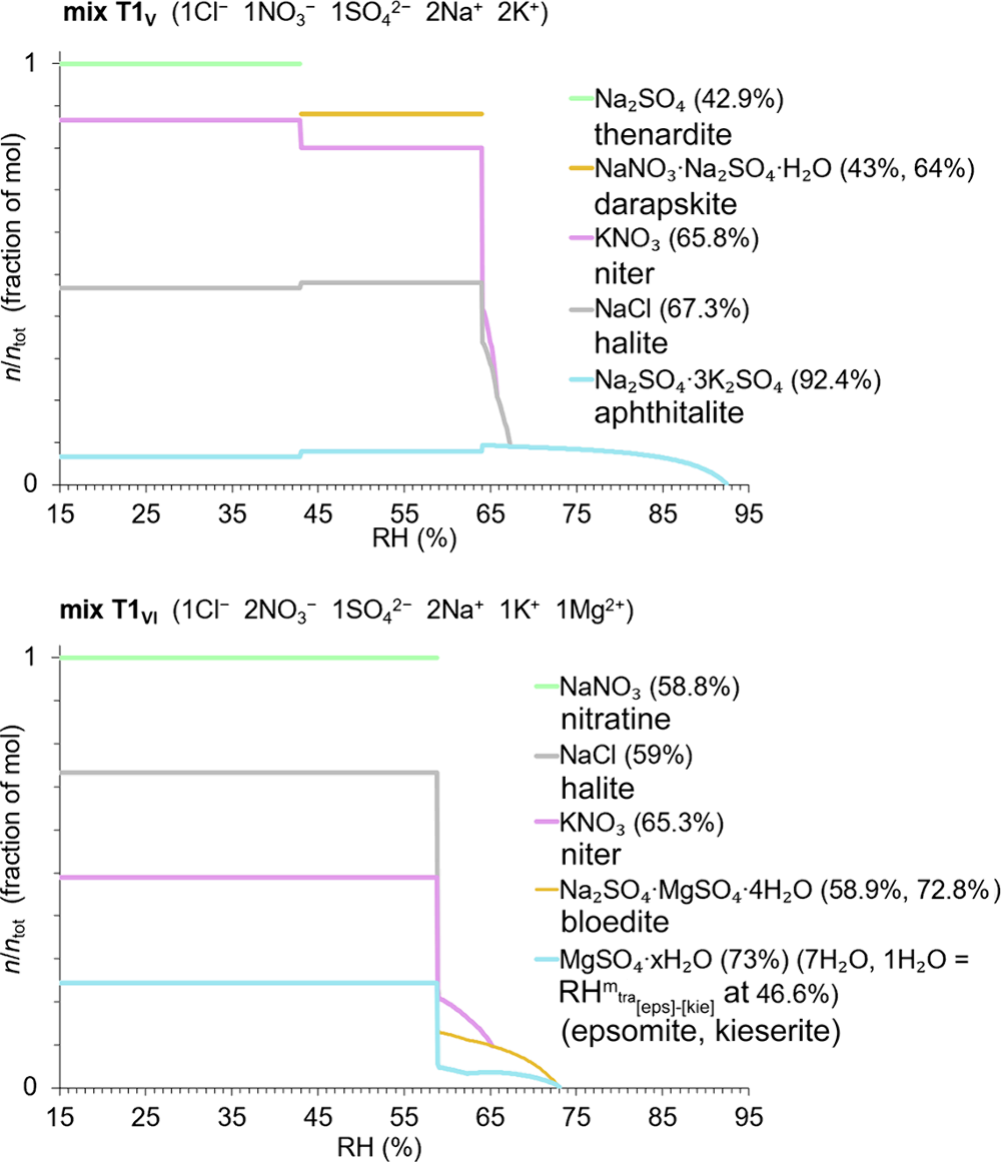
Crystallization and dissolution behavior showing solid phases of the type 1 salt mixtures and their mutual crystallization relative humidity (%), as modeled by ECOS/RUNSALT.^27,28^ Model limitations for magnesium sulfate hydrates are considered, as detailed in^22^. The *y*-axes depict crystalline solid as a fraction of mol in a stacked format, calculated at 20 °C, across 15–95% RH (*x*-axes: with 0.1% resolution). See Table 2 (method A) for an overview of RH points of interest.

**Figure 4:**
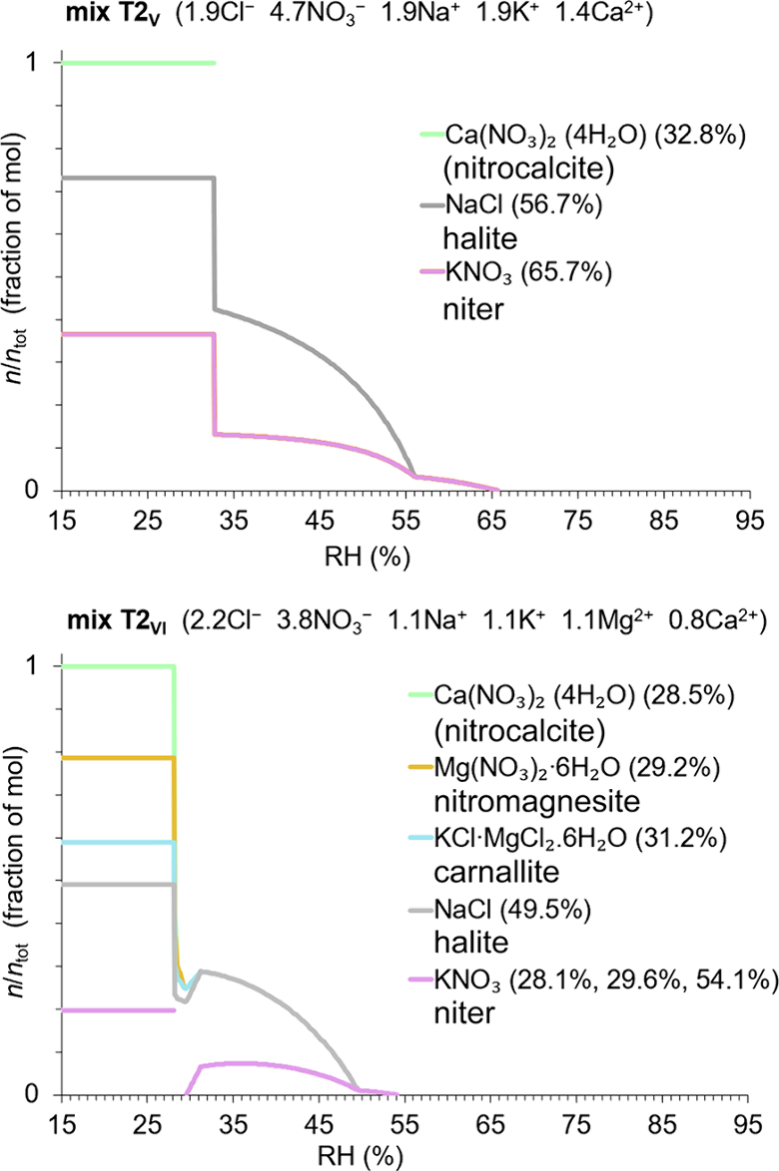
Crystallization and dissolution behavior showing solid phases of the type 2 salt mixtures and their mutual crystallization relative humidity (%), as modeled by ECOS/RUNSALT.^27,28^ Model limitations for calcium nitrate hydrates are considered, as detailed in^22^. The *y*-axes depict crystalline solid as a fraction of mol in a stacked format, calculated at 20 °C, across 15–95% RH (*x*-axes: with 0.1% resolution). See Table 2 (method A) for an overview of RH points of interest.

The modeled crystallization behavior of the four common mixtures (T1_V_, T1_VI_, T2_V_, and T2_VI_), as derived from the ECOS/RUNSALT model are shown in Figures 3 and 4. For a detailed explanation on how to interpret the plots, the terms, limitations, and solutions considering the model in- and output can be found in.^22,27,28^ In mixture type 1 (T1_V_ and T1_VI_), the solid phases that typically crystallize include aphthitalite, halite, niter, darapskite, thenardite, magnesium sulfate hydrates, bloedite, and nitratine. In these sulfate-enriched mixtures, most solid phases tend to crystallize around 65 and 60% RH for the five- and six-ion mixtures, respectively. An important note here is that the transition to thenardite in mix T1_V_ is a solid-state reaction (see^22^), which is not likely to be observed in the experimental results described further.

On the other hand, for mixture type 2 (T2_V_ and T2_VI_), frequently occurring solids are niter, halite, nitrocalcite, carnallite, and nitromagnesite. In these calcium-rich mixtures, a wider range of mutual crystallization RH is often observed between 65 and 30% RH. Notably, significant crystallization activities are commonly found under extremely dry conditions, below 35% RH, while anhydrous calcium nitrate is not stable,^7,32^ which is an identified issue in the model. Additionally, the dissolution of niter under drying conditions and its recrystallization as seen in mix T2_VI_ has also been observed in mix T2_V_, as further detailed in.^33^

### Time-Lapse Micrographs under Rapid Changing RH

**Figure 5:**
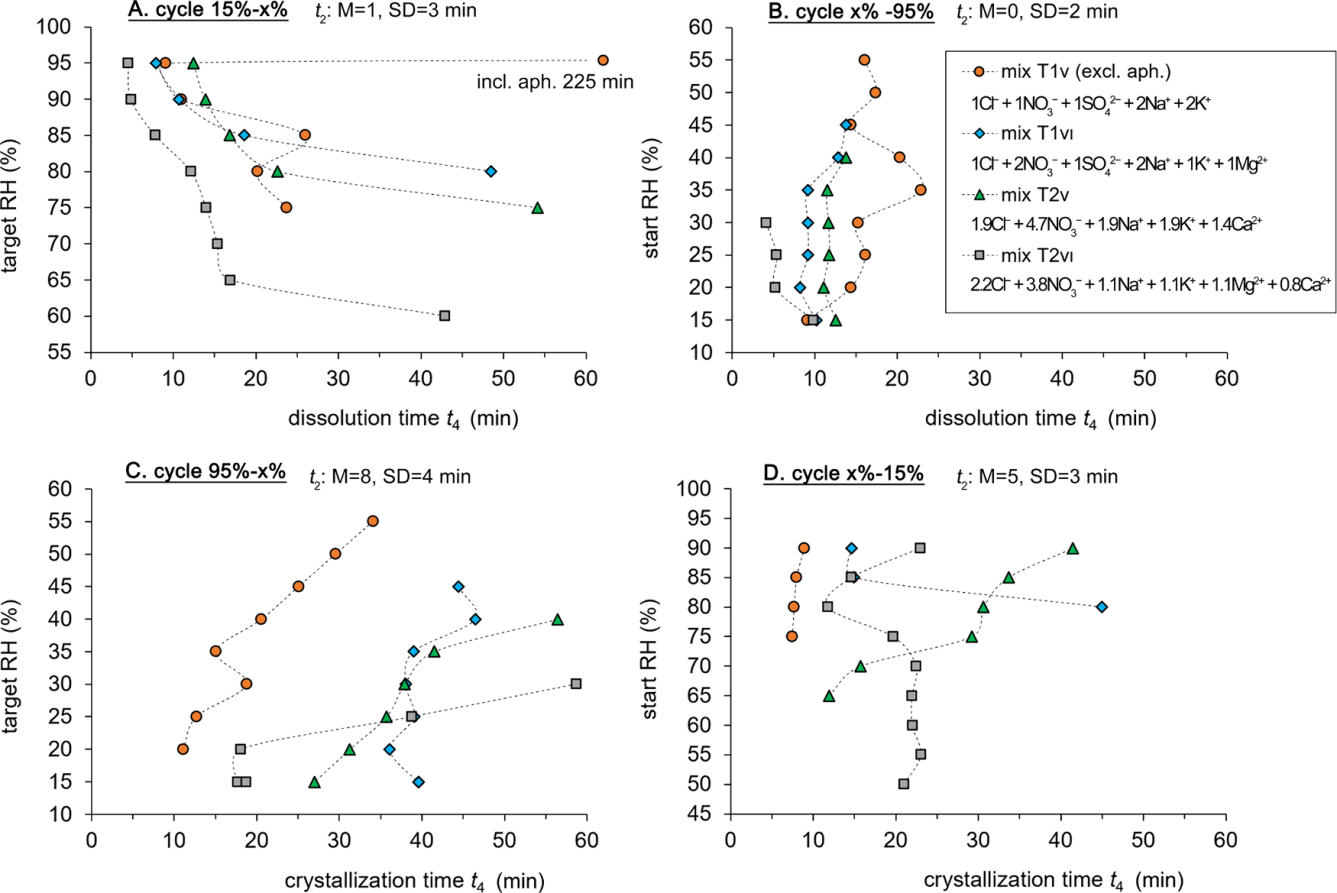
Data plots based on the analysis of the time-lapse micrographs identifying dissolution and crystallization processes for four mixture compositions (T1_V_, T1_VI_, T2_V_, and T2_VI_) under variable RH. The *x*-axes show time in minutes *t*_4_, equivalent to *t*_2_ (induction time) + *t*_3_ (completion time). Median (*M*) and standard deviation (SD) for *t*_2_ is specified. (A) and (B) show dissolution times for cycles from 15 to *x*% and from *x* to 95% RH, respectively. (C) and (D) depict crystallization times for cycles from 95 to *x*% and from *x* to 15% RH, respectively. Only complete processes are included. Aphthitalite in mixed T1_V_ is excluded due to dissolution times exceeding the 60 min RH step. Complete crystallization in mixes T2_V_ and T2_VI_ is also excluded due to kinetically hindered calcium nitrate crystallization.

The time-lapse micrographs (Figure 5A,B) illustrate the dissolution and crystallization times of salt mixtures under rapidly changing relative humidity conditions (0.6% s^–1^). For dissolution, the mixtures show a rapid onset (*t*_2_) and faster completion with increasing RH (mean time, *t*_3_: 10 min, SD = 3). However, when the RH target approaches the modeled mutual crystallization RH, a significant increase in dissolution time is observed with a mean *t*_4_ at 49 min and up to 225 min when considering aphthitalite in a mix of T1_V_. Interestingly, little influence is noticed on the dissolution times considering the crystal habit in all mixtures, specifically when comparing the dissolution of bulk crystals that are formed when the RH is set closer to the crystallization relative humidity or smaller crystals formed at lower RH (detailed further below). The crystallization times of the salt mixtures also show significant variations under rapidly changing RH conditions (Figure 5C,D). For crystallization, in mix T2_V_, a gradual increase in time to onset (*t*_2_) and completed crystallization (*t*_3_) was observed as the start RH is increased, ranging from 2 to 7 min and 12 to 41 min, respectively (Figure 5D). In contrast, a gradual decrease in time to onset is observed with 15 to 7 min and completion at 56 to 27 min when the target RH is increased (Figure 5C). Mix T1_V_ showed faster crystallization times, especially at lower RH targets, with onset times (*t*_2_) as low as 0 min due to the continuous presence of aphthitalite (not shown). The more hygroscopic calcium-rich mixtures (T2_VI_ and T2_V_) generally required longer times for crystallization completion, particularly when the RH was reduced from 95% to lower levels, reaching up to 59 min in mix T2_VI_, excluding crystallization of calcium nitrate (Figure 5C). The sulfate-rich, less hygroscopic mixtures (T1_V_ and T1_VI_), on the other hand, showed fast crystallization for T1_V_ and slower crystallization for T1_VI_, especially at RH levels of 15% (Figure 5D).

Completed dissolution processes are mostly in agreement with the modeled outputs for less hygroscopic sulfate-rich mixtures, this occurred above 75 and 95% RH for T1_V_, respectively, excluding and including aphthitalite, and above 80% RH for T1_VI_ (Figure 5A). For the more hygroscopic, calcium-rich mixtures, the observations deviate more from the model with completed dissolution above 75% RH for T2_V_ and above 60% RH for T2_VI_ (Figure 5A). These results indicate that completed dissolution processes under rapid RH changes occur at least 5% above the modeled dissolution RH. A notable deviation is seen for mix T2_V_, where this occurs approximately 10% RH above the indicated mutual crystallization relative humidity of the niter (65.7%) (Figure 5A). In contrast, completed crystallization processes diverge further from the model outputs likely due to supersaturation, which is a key factor influencing the kinetics. When a solution is supersaturated, it holds more dissolved material than what is predicted by thermodynamics at a given temperature and RH. Furthermore, the experimental parameters, particularly the rate of RH change, are linked to the observed RH at which crystallization occurs. In the less hygroscopic sulfate-rich mixtures, first completed crystallization is observed at 55% RH for T1_V_, excluding aphthitalite, and at 45% RH for T1_VI_ (Figure 5C). For more hygroscopic, calcium-rich mixtures, the first completed crystallization is seen from 40 and 30% RH for T2_V_ and T2_VI_, respectively (Figure 5C). Thus, deviating 10 to 25% RH from the modeled values at which all solids should crystallize, that is, under the experimental rate of RH decrease (0.6% s^–1^) and each RH step maintained for 1 h. This experimental setup intentionally does not always allow for the full induction time required for crystallization to reach completion to simulate the dynamic and varied climatic conditions encountered in real-world scenarios.

### Vapor Sorption under Slowly Changing RH

**Figure 6:**
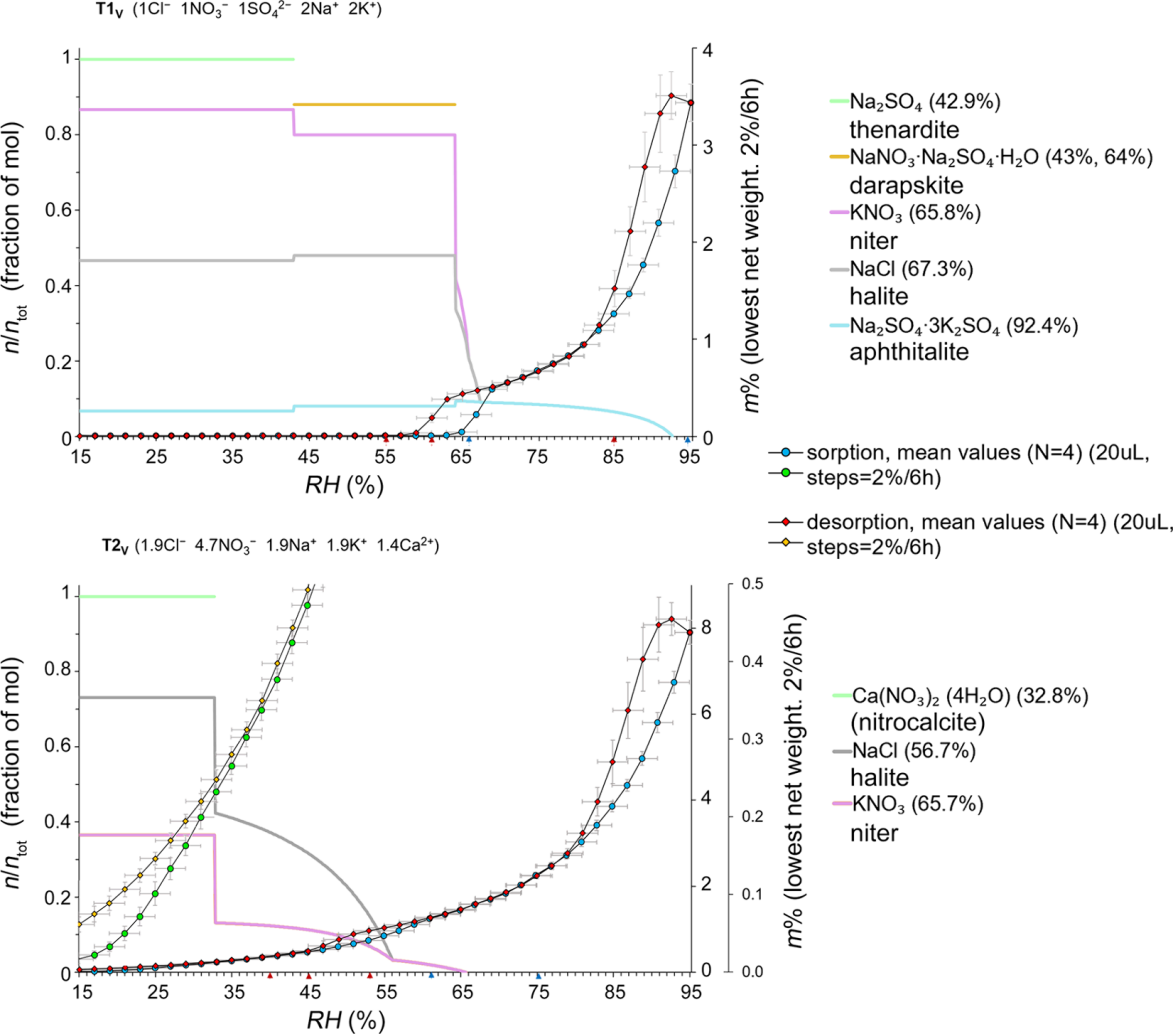
Crystallization behavior of five ion mixtures under slow RH changes (0.5% RH h^–1^) (maximum RH steps 2% per 6 h). T1_V_ (top) and T2_V_ (bottom). Calculations were performed using ECOS/RUNSALT at a temperature of 20 °C, with a 0.1% resolution. The primary *y*-axes show the modeled crystalline solid as a fraction of moles, presented in a stacked format, with the legend describing the various modeled solid phases. Secondary right *y*-axes display dynamic vapor sorption data (circles = sorption, diamonds = desorption) recorded at 20 °C in 2% RH intervals every 6 h (maximum time per step). The *x*-axes represent relative humidity (RH) ranging from 15 to 95%. The total time for sorption and desorption was 330 h. In the T2_V_ plot, sorption (green circles) and desorption (yellow diamonds) are displayed on a smaller scale (maximum 0.5 m%, tertiary *y*-axis) to highlight an otherwise invisible hysteresis loop (crystallization delay) occurring between approximately 30 and 15% RH.

As shown in the previous section, the rate of RH change has an impact on the crystallization and dissolution behavior of mixed ion solutions. Thus, the verification of the modeled behavior is further evaluated from sorption and desorption experiments under (equilibrium) conditions with steps of 2% each 6 and 50 h (maximum time). The mean results of four sorption and desorption measurements (2% each 6 h) of mix T1_V_ and T2_V_ are shown in Figure 6 in comparison to the modeled crystallization behavior. The results of T1_VI_ and T2_VI_ are similarly consistent with the modeled values as illustrated for T1_V_ and T2_V_ (refer to the Supporting Information). The initial hysteresis observed during the transition from sorption to desorption, starting from 95% relative humidity (RH) and decreasing, is attributed to kinetics; specifically, evaporation is slower than sorption and the measurement time is too fast for evaporation to achieve equilibrium conditions at the respective RH. The subsequent hysteresis demonstrates a similar phenomenon but is associated with the kinetics, most likely due to supersaturation of the solution, in relation to the rate at which the RH changes to achieve effective crystallization.

Specifically, the hysteresis at lower RH is attributed to crystallization kinetics and probable supersaturation, before crystallization, and the deviation of the branches is therefore useful to identify the critical crystallization humidity. Interestingly, when a slower experimental run is conducted (2% each 50 h), both forms of hysteresis become significantly less pronounced, thereby adding complexity to the interpretation. A logical phenomenon is that at high RH, more time is needed for evaporation to approach the equilibrium concentration. In our results, the hysteresis between sorption and desorption is clearly visible and in good agreement with the modeled values for mixtures T1_V_ (Figure 6, top) and T1_VI_ where the majority of salts crystallize. However, the crystallization of aphthitalite in T1_V_ cannot be determined from the mass changes alone, likely because the crystallization RH is too high. The results are also in close agreement with the modeled values for the more hygroscopic mixtures T2_V_ and T2_VI_, even though the hysteresis is less defined for these mixtures.

### Comparison between Modeled and Experimental Crystallization Behavior

To further identify the RH points of interest, the first derivative of the individual sorption and desorption measurements are calculated. The local maxima of the mean (*N* = 4 for 0.5% h^–1^ and *N* = 2 for 0.1% h^–1^) rate of change (first derivative) of sorption and desorption curves and the range of the hysteresis loops are summarized in Table 2. The table also presents the modeled (ECOS) RH points of interest for mutual deliquescence and crystallization. Additionally, it shows the mean (*N* = 2) RH points where these processes were observed in the micrographs from the sorption and desorption experiments carried out under rapid (GenRH) and slow (SPS) RH changes. The combined methods allow a detailed analysis of the kinetic processes. For both mixture types, sulfate-rich (T1_V_, T1_VI_) and calcium-rich (T2_V_, T2_VI_), deviations were observed between the modeled and the experimental RH points of interest.

**Table 2:** Summary of All RH (%) Points of Interest for Mutual Deliquescence and Crystallization as Modeled with ECOS/RUNSALT, Compared with the Results of the Experiments^a^

	**modeled**	**experimental**									
		**micrographs**						**mass change**			**Raman**
		rate of RH change at 20 °C									
**mixture**	RH^m^_del/cry_	0.6% s^–1^		0.5% h^–1^ (0.1% h^–1^)							0.5% h^–1^
**T1**_**V**_	92_aph_^b^	95	90		95 (93)	85 (89)			89	87 (85)	85
	67_hal_, 66_nit_, 64_dar_	75	55	66 (64)	(68)	61 (63)	56 (58)	69–57(67–61)	67 (65)	61 (63)	
**T1**_**VI**_	73_eps,blo_	80	45		81 (78)	53 (61)			75 (77)	53 (55)	
	65_nit_					(54)		79–45(75–53)	61 (60)		51
	59_hal,nitra_			61 (61)			45 (48)		49		
**T2**_**V**_	66_nit_	75	40		75 (72)	53 (53)		63–4	75 (77)	49 (53)	
	57_hal_				(57)				59 (55)		51
	33_nitro_			15			<15	33–15			
**T2**_**VI**_	54_nit_	60	30		63 (63)	44 (43)		53–37	49	39 (43)	
	50_hal_				(50)				36		39
	31_car_, 29_nit(ro)_			15	(33)	(23)	<15	37–15	15		
**method**	**A**	**B**	**C**	**D**	**E**	**F**	**G**	**H**	**I**	**J**	**K**

a The recorded RH points (mean *N* = 2) for dissolution and crystallization are determined from the micrographs under rapid (0.6% s^–1^) and slow (0.5% h^–1^ and 0.1% h^–1^) RH changes. With RH steps of 5% and 2%, each RH step was conditioned for either 1 h or a maximum of 6 and 50 h, for the rapid and slow runs, respectively. Furthermore, the mean (*N* = 4) RH ranges of the hysteresis loops between sorption and desorption curves are shown, as well as the maxima (max.) from the first derivative calculation of each individual curve and RH at which a change in wavenumber is identified in the Raman spectra (slow RH changes).

b Empty cells, unidentified. **A**: modeled mutual (m) crystallization (cry)/deliquescence (del) relative humidity (RH). **B**: completed dissolution observed at given RH. **C**: first crystallization observed at given RH. **D**: first dissolution observed at given RH during sorption. **E**: completed dissolution observed at given RH during sorption. **F**: first crystallization observed at given RH during desorption. **G**: completed crystallization observed at given RH during desorption. **H**: RH range of a hysteresis loop between sorption and desorption curves. **I**: first derivative maxima at given RH identified from the sorption curve. **J**: first derivative maxima at given RH identified from the desorption curve. **K**: RH when a change in wavenumber is identified in the Raman spectra. aph: aphthitalite, hal: halite, nit: niter, dar: darapskite, eps: epsomite, blo: bloedite, nitra: nitratine, nitro: nitrocalcite, car: carnallite.

These deviations are primarily attributed to the kinetic processes not considered in the modeled values. The experimental data allow us to better understand these processes under realistic changes of humidity. Here, we compare the first modeled mutual crystallization relative humidity (first ) with the initial crystallization RH observed in the micrographs under rapid and slow RH changes. Under rapid RH changes (0.6% s^–1^), crystallization in T1_V_ occurred 2% (incl. aphthitalite) and 12% (excl. aphthitalite) below the modeled values. For T1_VI_, the deviation was 28%, while for the calcium-rich more hygroscopic mixtures, the deviations below the modeled values are 26% for T2_V_ and 24% for T2_VI_. The differences under slow RH changes (0.5% h^–1^) are 7 and 6% for T1_V_, including and excluding aphthitalite. For T1_VI_, they are 20%, while for T2_V_, they are 13 to 21%, and finally for T2_VI_, a 10% deviation was observed.

As expected under slower RH changes (0.1% h^–1^, desorption), initial crystallization was observed closer to the modeled values deviating only 3 and 4% for T1_V_, including and excluding aphthitalite. While a 12% deviation was observed for T1_VI_, the slower process also allowed an additional identification of niter crystallization 11% below the modeled value. Interestingly, for the more hygroscopic mixtures, the deviations were similar under both 0.5 and 0.1% h^–1^, with 13 and 11% for respectively T2_V_ and T2_VI_. Again, for T2_VI_, an additional process was observed at 8% below the . We can reasonably state that crystallization consistently occurs at lower RH due to a kinetic delay, likely caused by the supersaturation of the solution in relation to the rate of change, as further described in^26,34−38^.

When investigating the dissolution behavior, initial dissolution RH values are closely in agreement with the modeled values for the sulfate-rich mixtures (T1) while significant deviations are observed for the hygroscopic T2 mixtures. The latter is related to the kinetically hindered crystallization of calcium nitrate and the continuous presence of solution throughout the experiment. For the completed dissolution processes (sorption), the deviations remain under 10% for slow (0.5% h^–1^) and rapid (0.6% s^–1^) humidity changes with a mean value of approximately 7% above the last modeled solid in solution, that is, the first mutual crystallization RH. In comparison, under slower conditions (0.1% h^–1^), the mean deviation was 3%, thus allowing for a higher accuracy, validating the modeled values.

Under rapid RH changes (0.6% s^–1^), completed dissolution was observed 3 and 8% above the modeled values for T1_V_, respectively, excluding and including aphthitalite. Similar deviations were recorded under a slow rate of the RH change (0.5% h^–1^). For T1_VI_, the deviations are 7 and 8%, respectively, for rapid and slow changes while for T2_V_ and T2_VI_, 9 and 6% are seen under both rates of change. The difference is less pronounced under slower conditions (0.1% h^–1^), yet they remain significant for the calcium-rich mixtures (T2). A summary of the deviating RH values specific to each mixture type under rapid or slow RH changes is given in Table 3. Notably, initial dissolution was observed at the same RH (±2%) as modeled for the sulfate-rich mixtures (T1), while the deviation was at least 15% for the calcium-rich mixtures (T2) due to the kinetically hindered crystallization of calcium nitrate.

**Table 3:** Summary of the Experimental Results Considering the Kinetics of Common Mixtures under Realistic Humidity Rate Changes^a^

	**modeled**		**experimental^b^** −RH=							
	1^st^ RH^m^_cry_*x*__	RH^m^_del_*x*__	1^st^cry–1^st^RH^m^_cry_*x*__			1^st^dis–RH^m^_del_*x*__		c.dis–1^st^RH^m^_cry_*x*__		
			rate of RH change at 20 °C							
**mixture**			0.6% s^–1^	0.5% h^–1^	0.1% h^–1^	0.5% h^–1^	0.1% h^–1^	0.6% s^–1^	0.5% h^–1^	0.1% h^–1^
**T1**_**V**_	92_aph_		–2	–7	–3			+3	+3	+1
	67_hal_	64_dar_	–12	–6	–4	+2	0	+8		+1
**T1**_**VI**_	73_eps,blo_	59_hal,nitra_	–28	–20	–12	+2	+2	+7	+8	+5
**T2**_**V**_	66_nit_	33_nitro_	–26	–13	–13	–18		+9	+9	+6
**T2**_**VI**_	54_nit_	29_nitro_	–24	–10	–11	–12		+6	+9	+9
mean ΔRH			–18	–11	–9			+7	+7	+3

a The RH (%) deviations are shown between modeled and experimental RH points of interest (ΔRH), specifically the modeled mutual crystallization and mutual deliquescence RH compared to the observed RH values at which first crystallization and (first and completed) dissolution occurred in the different mixture types under rapid (0.6% s^–1^) and slow (0.5% h^–1^ and 0.1% h^–1^) RH changes.

b Empty cells, observations, and experimental data were inconclusive, and processes could not be observed. The RH steps under the rate of change are ±5 and ±2% for 0.6% s^–1^ and (0.5% and 0.1 h^–1^), respectively. m: mutual, cry: crystallization, del: deliquescence, dis: dissolution, c.dis: completed dissolution (all solids are dissolved), *x*: associated solid, aph: aphthitalite, eps: epsomite, hal: halite, dar: darapskite, blo: bloedite, nitra: nitratine, nit: niter, nitro: nitrocalcite.

As shown by the difference between RH values at which crystallization and dissolution occur when compared to the modeled values, the obtained sorption data (dissolution) allow for a more accurate indicator of , also described by^39^. When salt crystals dissolve, the water activity of the solution increases, accounting for the steeper slopes during the sorption run. However, in complex mixtures, specifically ones with extreme hygroscopic properties (T2 mixtures), the mass change becomes negligible between solid and solution under increasing RH; as the former dissolves, the latter picks up water molecules accounting for the mass loss. Thus, we explain the discrepancies between the RH values determined via the sorption data and the micrographs.

The sorption and desorption curves obtained remain important to identify critical RH ranges in which the solids crystallize, determined from the first derivative (maxima) of sorption and the hysteresis loops (Table 2). The RH values identified are highly accurate for T1 mixtures, considering a 2% experimental resolution. These results are also in good agreement with the observations of the first visible dissolution. Especially, for T2 mixtures, deviations are recorded, with the modeled  at 66% compared to the first derivative of sorption at 75% for T2_V_ and from modeled  at 54 to 49% for T2_VI_. However, the RH ranges of the hysteresis loops closely align with the majority of processes for all investigated mixtures, also showing crystallization delays between approximately 35 and 15% RH for more hygroscopic mixtures (T2). The latter is further illustrated with the sorption and desorption curves displayed on a smaller scale to highlight an otherwise invisible hysteresis loop (crystallization delay) occurring between approximately 30 and 15% RH, as displayed for T2_V_ in Figure 6. Particularly, the behavior of KNO_3_ in T2_V_ showed a clear deviation, suggesting that model parameters may require refinement when K^+^, NO_3_^–^, and Ca^2+^ coexist in a mixture. It remains important to note that the model can be considered highly accurate and that the crystallization delays observed in both mixture types can primarily be attributed to kinetic delays.

### Identification of Salt Phases and Investigating Crystal Habit

The RH values at which crystallization and dissolution occur throughout the SPS measurements are further validated with the Raman spectra recorded at approximately each 5% RH step throughout the SPS desorption measurements (refer to the Supporting Information). The first observed crystallization in the mixtures coincided with a change in the wavenumber (cm^–1^) identified in the Raman spectra. Initial crystallization was confirmed at 85, 51, 51, and 39% RH for T1_V_, T1_VI_, T2_V_, and T2_VI_, respectively. The results are closely in agreement with the observations of first crystallization, as identified in micrographs throughout the SPS experiments. Specific bands are closely related to aphthitalite and niter in T1_V_, nitratine, magnesium sulfate hydrates, arcanite, and niter in T1_VI_, while niter was identified in T2_V_ and T2_VI_. The Raman identification of solids during the faster GenRH experiments were carried out with higher resolution, thus allowing additional identification of darapskite, niter, and aphthitalite for T1_V_, magnesium sulfates, nitratine, and niter for T1_VI_, while niter and traces of nitrocalcite were identified in both T2_V_ and T2_VI_ including nitratine in the latter. The results confirm the modeled results, while taking the limitations of thermodynamic calculations into account.

**Figure 7:**
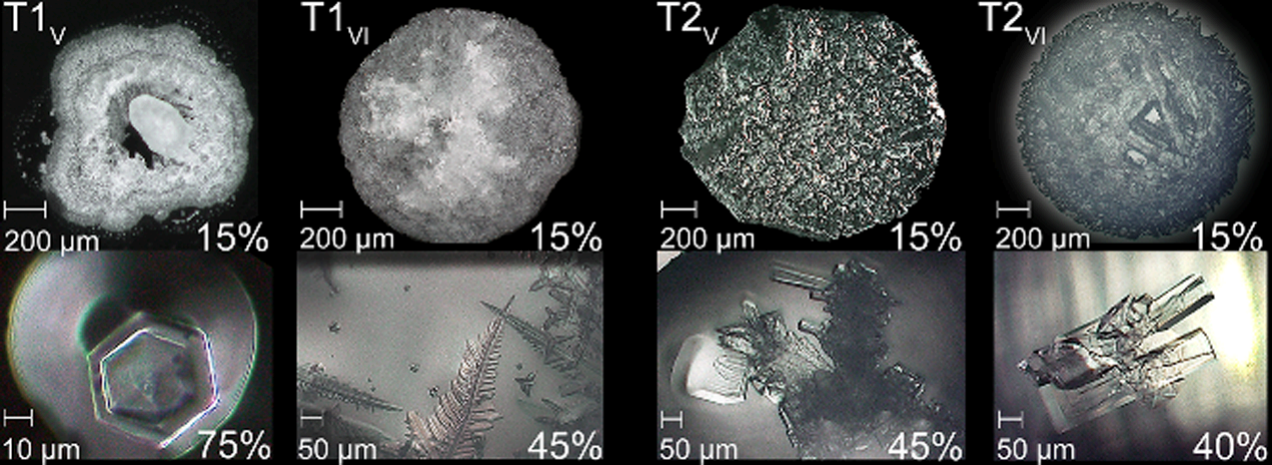
Illustration of crystal habit identified in the micrographs under changing RH conditions and 20 °C for all investigated mixtures, from left to right T1_V_ to T2_VI_. The top images show the crystallized solutions (initial volume 0.5 μL) after rapid RH decrease (approximately 0.6% s^–1^) from 95 to 15% RH. The bottom images example specific crystal habit, from left to right: first, hexagonal prism-shaped crystal associated with aphthitalite. Second, fern-like dendritic crystals were identified as niter. Third, a clump of aggregated crystals with a mix of shapes, some of which have a tubular or rod-like morphology (niter), emerging from a central core and cubic shape associated with halite, and last, elongated tubular and cubic (hopper) crystals with multiple facets (niter and halite). Solution remained available in calcium-rich mixtures (T2) at 15% RH.

Additional XRD analysis was carried out on the dried solutions (refer to the Supporting Information). Results were obtained for both sulfate-rich (T1) mixtures. As modeled by ECOS/RUNSALT, darapskite, aphthitalite, niter, and halite were identified in the dried mixture T1_V_. As expected, thenardite was absent, as its formation in this mixture is the result of a solid-state decomposition reaction of darapskite and aphthitalite, which had not occurred within the experimental time frame. This result validates the statement in^22^, showing that solid-state reactions might have a limited effect on porous materials under daily changes in RH. The mixture T1_VI_ showed the presence of starkeyite, nitratine, halite, and niter. Although the formation of starkeyite was not modeled, an issue with magnesium sulfate hydrates in ECOS was expected, as detailed in^25^. In both cases, the XRD results are in agreement with the ECOS modeled solids. On the other hand, despite the long-term conditioning of the calcium-rich (T2) mixtures, the XRD analysis was unable to identify any minerals. This was attributed to persistent calcium nitrate solution surrounding all solids, which had become extremely viscous and exhibited amorphous properties, thus obscuring the definitive crystalline characteristics required for identification (Figure 7, top T2_V_ and T2_VI_).

**Figure 8:**
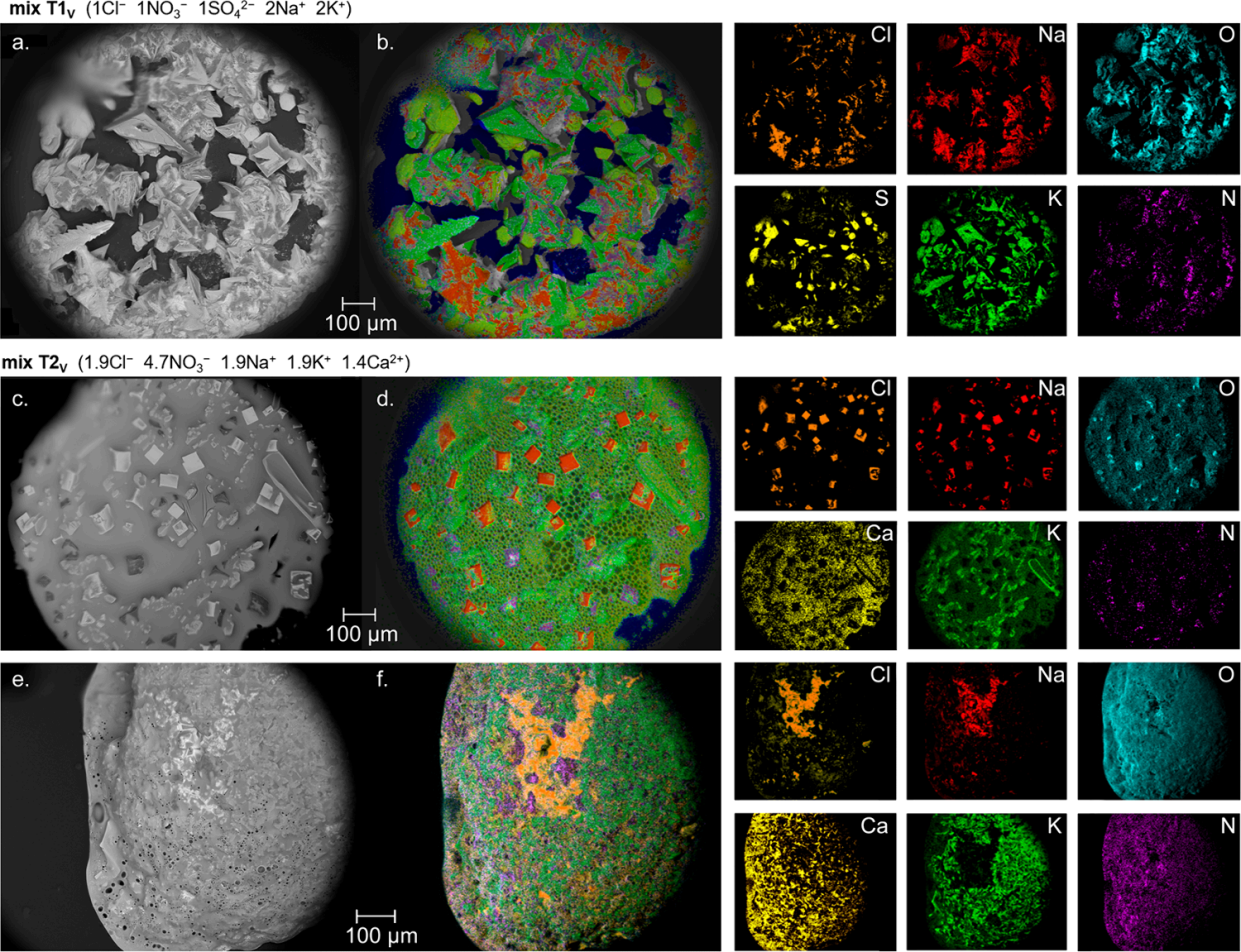
Illustration of crystal habit ESEM image (left a, c, e) and layered EDX image (b, d, and f) including element distribution (K-series) on the right for mixture T1_V_ (a, b) 1Cl^–^ + 1NO_3_^–^ + 1SO_4_^2–^ + 2Na^+^ + 2K^+^, and for mixture T2_V_ (c, d) 1.9Cl^–^ + 4.7NO_3_^–^ + 1.9Na^+^ + 1.9K^+^ + 1.4Ca^2+^, after a slow evaporation rate, while panels e and f show the same mix T2_V_ after a fast evaporation rate. Images were obtained between 0 and 1.2% RH and 5 °C (vacuum settings 9.79 × 10^–4^ or 10 Pa) after decreasing the chamber pressure in steps of 20% RH each hour from 95 to 15% (slow) or from solution directly under high vacuum (fast), at 5 °C. Note that solution remains available in mix T2 under both rates of evaporation at 0% RH. The bubbles in (d) are boiling solution caused by beam heating at the surface.

To further aid the identification of salts and the possible relation with stone decay associated with crystal size (see refs (8,40,41)), the habit of crystals was also investigated via (E)SEM-EDX (Figure 8). The evaporation rate and surface tension have significant influence on crystal habit as described in,^42−44^ which implies different degrees of (super)saturation.^36,37,45^ Additionally, it has been shown that a salt that crystallizes from a mixture has smaller dimensions when compared to its crystal size from a less complex ion solution;^46^ more specifically, crystal size is reduced in mixtures compared to single salt dimensions. Many different habits were identified in the micrographs, most notably typical hexagonal structures and semispherical platy aggregates of aphthitalite, cubic, and hopper crystal systems related to halite and sylvite, and orthorhombic crystals, that is, long prismatic shapes with needle-like or plate-like forms identified as niter.

Other habits identified were different types of polyhedral crystals with typical flat faces (facets) and sharp angles. Additionally, dendritic and needle morphologies were identified in all four mixtures that were mainly related to niter. The more robust formed crystals were primarily obtained when the RH target was nearer to the critical crystallization RH of the related solid (for example Figure 8a,c). On the other hand, we observed more dendritic, microcrystalline, disordered clusters and amorphous structures in the experiments where the rate of change is rapid and the RH target is further away from the critical crystallization RH (for example, Figure 8e). Under these conditions, polycrystalline dendritic and microcrystalline patterns formed around the initial bulk, often out of the last remaining solution and sometimes growing up to three times the distance over the surface beyond the initial droplet circle.

The identification of these crystal habits and element distribution further validated the model calculated solids, including clear identification of clusters related to sodium chloride (halite), potassium nitrate (niter), sodium potassium sulfate (aphthitalite), and calcium nitrate in solution (for example, Figure 8b,d,f and element distributions on the right).

## Conclusions

Understanding the kinetics of salt mixtures under varying relative humidity (RH) conditions is important for applications ranging from built heritage conservation to geological investigation. This research combined several methods, including time-lapse micrographs and dynamic vapor sorption, to explore changes in humidity conditions ranging from 15 to 95% RH (at 20 °C). The behavior of salt mixtures, frequently identified in the built environment, was established. These mixtures are categorized into two types: type 1 (sulfate-rich) and less hygroscopic and type 2 (calcium-rich) and more hygroscopic. Each mixture contains five or six ions: Cl^–^, NO_3_^–^, Na^+^, K^+^, and either SO_4_^2–^ or Ca^2+^, with Mg^2+^ present as the sixth less common ion. To mimic realistic climate scenarios, different rates of RH changes were subjected to the mixtures (droplets), with rapid changes (0.6% s^–1^ = approximately 80% RH change within 133 s) and slow changes (0.5% h^–1^ = approximately 80% RH change over 160 h). Additionally, even slower experiments (0.1% h^–1^) were carried out to verify modeled RH points of interest and optimize the method. These selected RH changes aim to represent approximately 80% RH change over varying periods: minutes, a week, and up to a month.

Various analytical techniques were utilized to verify phase transitions and crystal habits associated with different RH conditions, including environmental scanning electron microscopy (ESEM), micro-Raman spectroscopy, X-ray diffraction (XRD), and elemental mapping via energy-dispersive X-ray spectroscopy (EDX). The behavior of the mixtures as modeled (ECOS/RUNSALT) was verified against experimental observations, confirming the model’s accuracy but also showing significant deviations mainly attributed to kinetic factors, such as supersaturation. This reveals the necessity of kinetic considerations in future models and risk assessments. Despite expectations of kinetic variations during desorption processes, the discrepancies between sorption or dissolution measurements and theoretical calculations indicate that parameters, especially those related to the calcium-rich (hygroscopic mixtures), need further examination. The study’s results illustrate a relationship between the kinetics of phase transitions and changes in RH, with the onset of crystallization and dissolution showing mean deviations from modeled expectations. Interestingly, the rates of RH change had a minor influence on these deviations, suggesting a slight unresponsiveness to different environmental change rates. The practical implications of these findings are significant for both built heritage conservation and geological studies, enabling a more precise approach to in situ preservation strategies and the prediction of salt deposition and dissolution mechanisms. These insights are essential for future modeling efforts to address complex phenomena in both built and natural environments. However, additional parameters, such as different temperatures, water, wind, solar radiation, in-pore processes, and changes in mixture composition, need consideration.

Furthermore, the innovative approach of combining analytical techniques has highlighted important kinetic delays in crystallization and dissolution compared to modeled behavior. Especially for type 2 (calcium-rich) mixtures, which remained in solution throughout the experiments under extreme dry conditions, factors such as kinetically hindered crystallization, delay effects due to the rate of supersaturation, and dissolution delays caused by a combination of concentration gradients, surface tension, water activity, crystal microstructure, and surface characteristics indicate the dynamic and complex nature of these processes. These findings underscore the need for further fundamental research to understand the impact of these factors on crystal behavior more comprehensively. Overall, the insights gained from this study have broad implications for forming conservation management strategies, especially in the context of historical monument preservation. The detailed examination of how salt mixtures respond to varying RH conditions contributes valuable knowledge to the field, highlighting the importance of using combined analytical techniques and the need for multifactorial models in environmental conservation and historical preservation planning.

## Data Availability

The raw data is stored on the internal servers of the Royal Institute for Cultural Heritage (KIK-IRPA, Jubelpark 1, 1000 Brussels, Belgium) under the Open Science Mandate of the Belgium Science Policy (Belspo) and is available at reasonable request to the corresponding author (SG) or via info@kikirpa.be.

## Supporting Information Available

Raman reference spectra (in-house library), Raman spectra obtained throughout the desorption measurements at different RH values at 20 °C, and XRD spectra obtained for mixtures T1_V_ and T1_VI_ (PDF)
Comparative data for mixtures T1_V_, T1_VI_, T2_V_, and T2_VI_; ECOS/RUNSALT raw data and outputs, selected sorption and desorption data and graphs; selected micrographs of crystallization and dissolution processes; and XRD analysis, Raman, and ESEM-EDX analysis data and figures (XLSX)

## Abbreviations

T1_V_, T1_VI_, T2_V_, T2_VI_: mixture types, _V_: five ions or _vi_: six ions
T1: type 1 mixture (sulfate-rich) Cl^–^, NO_3_^–^, SO_4_^2–^, Na^+^, K^+^, (Mg^2+^)
T2: type 2 mixture (calcium-rich) Cl^–^, NO_3_^–^, Na^+^, K^+^, Ca^2+^, (Mg^2+^)
ECOS: environmental control of salts (thermodynamic model)
RUNSALT: user interface to the ECOS model (GUI)
GenRH: relative humidity generator (equipment name, Surface Measurements Systems)
MCell: small, windowed climate chamber connected to GenRH system (equipment name)
SPS: sorption testing system (equipment name, ProUmid), dynamic vapor (de)sorption (DVS)
ESEM: environmental scanning electron microscopy
EDX: energy-dispersive X-ray spectroscopy
K-series: X-ray emissions resulting from electron transitions to the K-shell in energy-dispersive X-ray spectroscopy (EDX) elemental analysis.
EHT: Extra High Tension
LaB6: lanthanum hexaboride, thermionic emitter in electron microscopes
NTS BSD: nanoTechnology Systems BackScattered detector
XRD: X-ray diffraction
RH: relative humidity
SD: standard deviation
h: hour
s: second
Pa: Pascal
cm^–1^: centimeter inverse (wavenumber in Raman spectroscopy)
mw: milliwatts
ms: millisecond
μL: microliter
μm: micrometer
sccm: standard cubic centimeters per minute
kg^–1^: kilogram inverse (molality)
*M*: mol ratio (SPS (DVS) measurements)
*R*: RH at the step (SPS (DVS) measurements)
*t*_1_: time from start of the RH step to first mutual crystallization or dissolution RH target
*t*_2_: induction time until first visible crystal or dissolution
*t*_3_: effective time for complete visible crystallization or dissolution
*t*_4_: *t*_2_+*t*_3_
*t*exp: total experimental time for each RH step
*M*: median
m: mutual
cry: crystallization
del: deliquescence
dis: dissolution
*x*: associated solid or hydrate
aph: aphthitalite
dar: darapskite
eps: epsomite
blo: bloedite
hal: halite
nitra: nitratine
nitro: nitrocalcite
nit: niter
car: carnallite
MgSO_4_·*x*H_2_O: magnesium sulfate hydrates
