# Supplementary material for: Modeled versus Experimental Salt Mixture Behavior under Variable Humidity
Source: ACS Omega. 2024 Mar 27;9(18):20454–66. doi: 10.1021/acsomega.4c01486 (PMC11080023; doi:10.1021/acsomega.4c01486)

Supplementary Information file

Modeled versus experimental salt mixture behavior under variable humidity

Sebastiaan Godts [1,2,3,*], Michael Steiger [4], Amelie Stahlbuhk [4], Scott Allan Orr [5], Julie Desarnaud [1], Hilde De Clercq [1], Veerle Cnudde [3,6] & Tim De Kock [2]

1 Monuments Lab, Royal Institute for Cultural Heritage (KIK-IRPA), Brussels, Belgium

2 Antwerp Cultural Heritage Sciences (ARCHES), University of Antwerp, Belgium

3 Department of Geology (PProGRess), Ghent University, Belgium

4 Department of Chemistry, University of Hamburg, Germany

5 Institute for Sustainable Heritage, University College London (UCL), United Kingdom

6 Department of Earth Sciences, Utrecht University, Utrecht, The Netherlands

* Corresponding author: Sebastiaan Godts (sebastiaan.godts@kikirpa.be)

ABSTRACT: This study investigates the kinetics of salt mixture crystallization under relative humidity (RH) conditions, varying between 15% and 95% (at 20 °C), to inform applications in built heritage preservation, geology and environmental sciences. We focused on commonly found, sulfate-rich and calcium-rich salt mixtures containing five to six ions, Cl^-, NO_3^- , Na^+, K^+, including or excluding less common Mg^{2+}, and including either an excess of SO_4^{2-} or Ca^{2+}, with respect to gypsum. Using time-lapse micrographs and dynamic vapor sorption, we explore how crystallization and dissolution behavior depends on RH and mixture composition under constant temperature. A range of RH change rates were studied to simulate realistic weather events. Microstructural analyses through Environmental Scanning Electron Microscopy (ESEM) confirmed crystal habit corresponding with RH transitions. Phases predicted from thermodynamic modeling (ECOS/RUNSALT) were confirmed using micro-Raman spectroscopy, X-ray Diffraction (XRD), and elemental mapping via Energy Dispersive X-ray Spectroscopy (EDX). We identify a strong correlation between phase transition kinetics and RH change rates, with crystallization deviating by -15% and dissolution by +7% from modeled values under rapid (several seconds) and slow (several days) RH changes. These insights are important for preservation strategies in built heritage, salt deposition and dissolution mechanisms in diverse geological and realistic environmental contexts, laboratory experiments, future modeling efforts and the understanding of stone decay in general.

Supplementary material 001

Table S1 : Raman Reference Spectra Table (in-house library)

Raman Shift, wavenumber (cm^{-1})	Chemical	Mineral
992.7	$K_3Na(SO_4)_2{\cdot}6H_2O$	aphthitalite
1068.1	$NaNO_3$	nitratine
997.0, 1063.4	$Na_3NO_3SO_4{\cdot}H_2O$	darapskite
983.6	KSO_4	arcanite
993.4	Na_2SO_4 (V)	thenardite
996.7	Na_2SO_4 (III)	
988.3	$Na_2SO_4{\cdot}7H_2O$	
989.8	$Na_2SO_4{\cdot}10H_2O$	mirabilite
719, 743, 1051.4, 1354, 1427	$Ca(NO_3)_2{\cdot}4H_2O$	nitrocalcite
715, 1050.3, 1344.5, 1359.5	KNO_3	niter
732, 1060.1, 1360.5, 1434	$Mg(NO_3)_2{\cdot}6H_2O$	nitromagnesite
433-482.5, 627.5, 1046, 1118.5, 1216.5	$MgSO_4{\cdot}1.25H_2O$	
277, 440-471, 614, 1047, 1116.5-1139, 1172.5-1217.5	$MgSO_4{\cdot}2H_2O$	
479.5, 1000.5, 1089, 1149	$MgSO_4$ $4H_2O$	starkeyite
467, 983.5	$MgSO_4{\cdot}6H_2O$	hexahydrite
450-462, 616, 984.5	$MgSO_4{\cdot}7H_2O$	epsomite
219-267.5, 432-500, 629.5-668, 1043.5, 1120, 1217.5	$MgSO_4{\cdot}H_2O$	kieserite
450-462, 634, 985.2, 1082.5	$K_2Mg(SO_2)_4{\cdot}6H_2O$	picromerite

Table S2 : Raman spectra obtained throughout the desorption measurements (SPS) at different RH values at 20 ° C. Legend: sol.=solution, cry.=crystallization, comp.=completed. Observations are related to micrographs obtained throughout the measurements.

mixture	RH (%)	Raman shift, wavenumber (cm^{-1})												Observations
$T1_V$	95 to 90						981	1050						sol.
	85 to 55	452	621			984	993	1050		1082				1st cry.
	51 to 29	452	621		710	720	993	1050	1067	1082			1385	2nd cry.
	25 to 19	452	621		710	730	993	1050	1067	1082			1385	comp. cry.
$T1_{VI}$	95 to 55						984	1050						sol.
	51 to 19	465	600	640	715	726	984	1050	1068	1147	1344	1359	1385	1st / comp. cry.
$T2_V$	95 to 55				715	739		1049			1340		1400	sol.
	51				715	739			1052		1340		1400	1st cry.
	47 to 19				715	739		1050	1052		1345		1361	2nd cry. (sol.)
$T2_{VI}$	95 to 43				719			1050	1052		1340		1400	sol.
	39				719				1052		1340		1400	1st cry.
	37 to 19				715			1050	1068		1344	1359	1385	2nd cry. (sol.)
resolution of the obtained wavenumbers: mean and median 1.7, min 1.2, max 2.3, SD 0.3 (all as cm^{-1})														

Figure S1 : Raman Reference Spectra Table (in-house library)

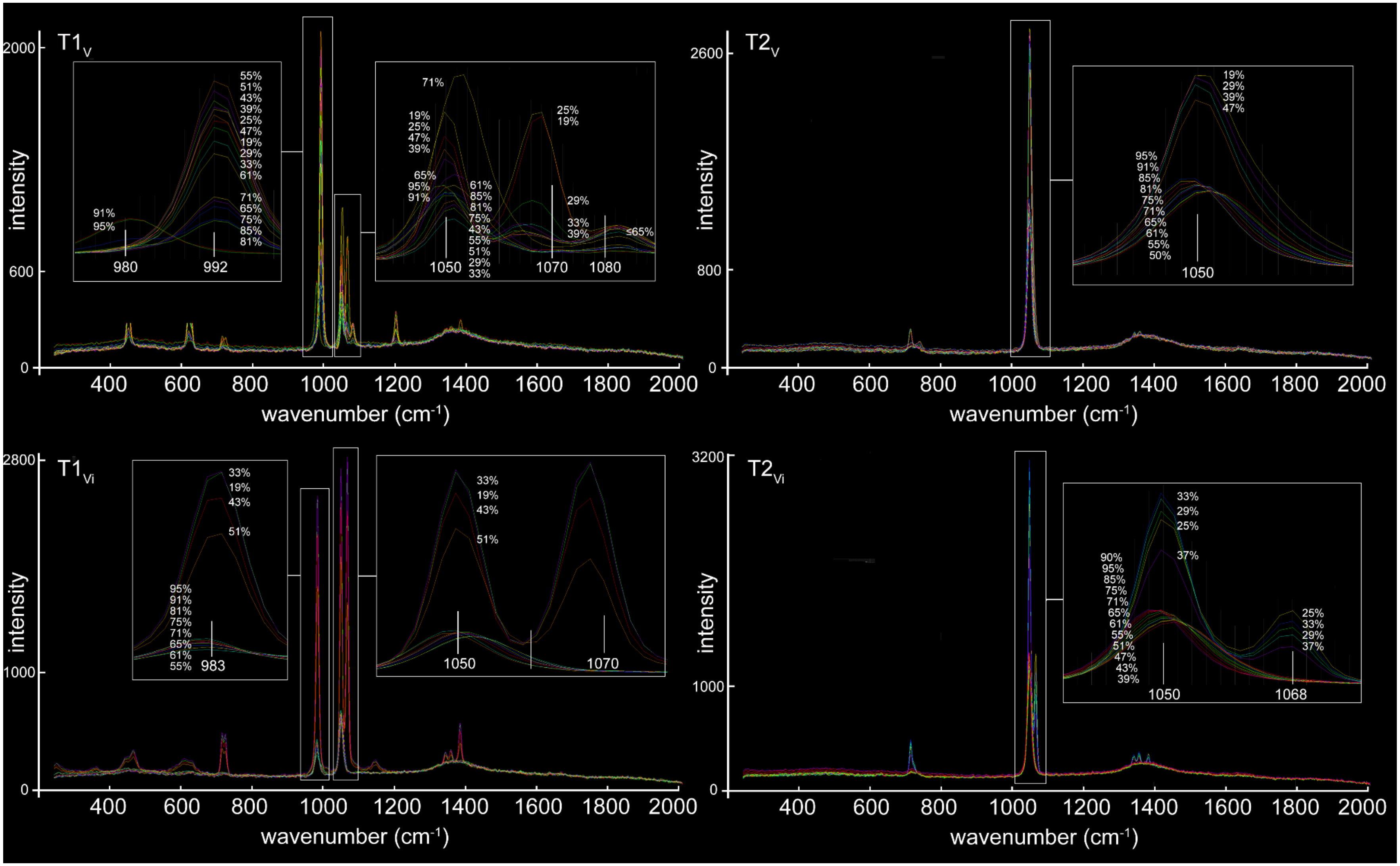

Supplementarymaterial 002

Figure S2 : XRD analysis mix T1V (1Cl– 1NO3– 1SO42– 2Na+ 2K+)

(Coupled TwoTheta/Theta)

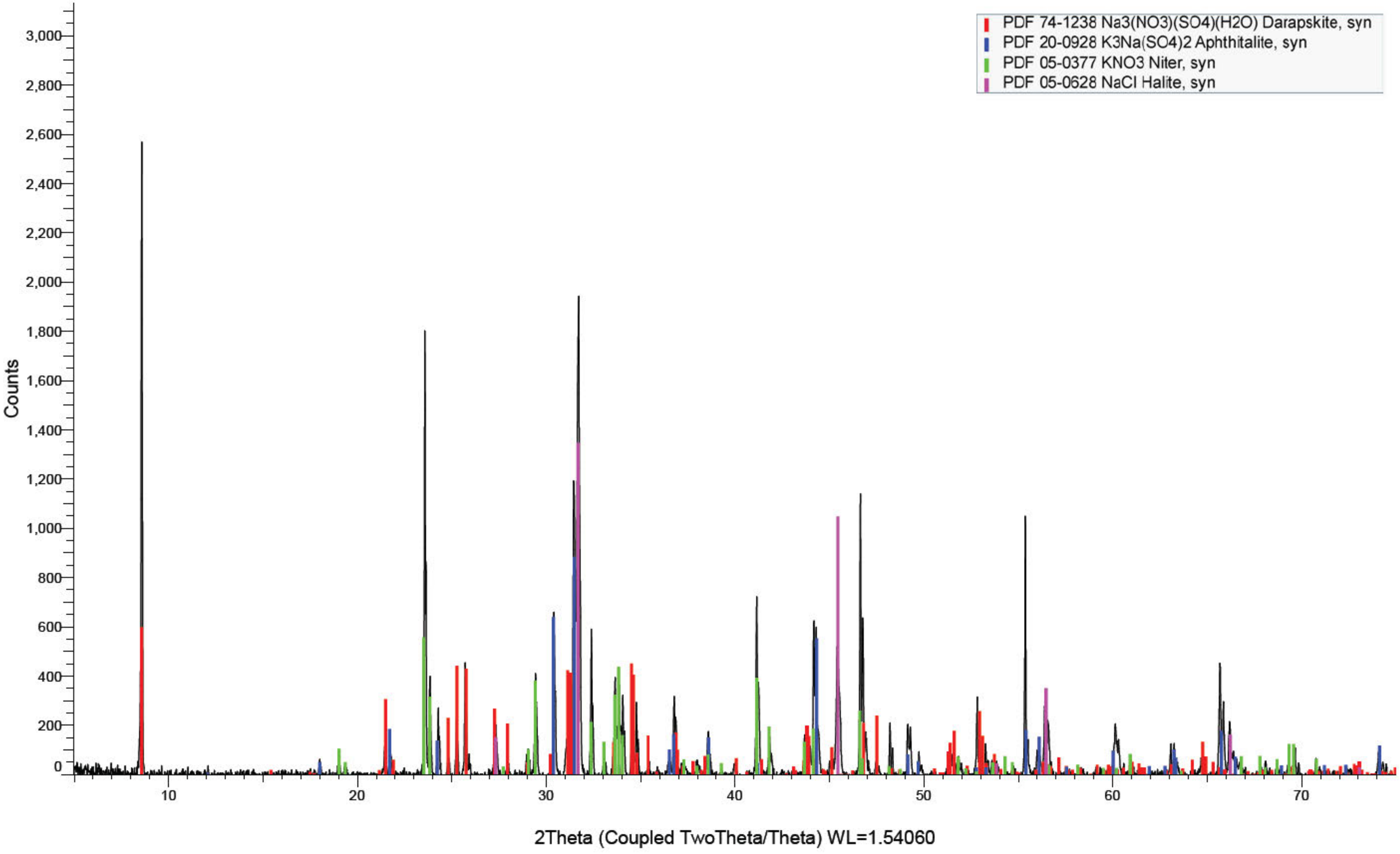

Figure S3 : XRD analysis mix T1VI (1Cl– 2NO3– 1SO42– 2Na+ 1K+ 1Mg2+)

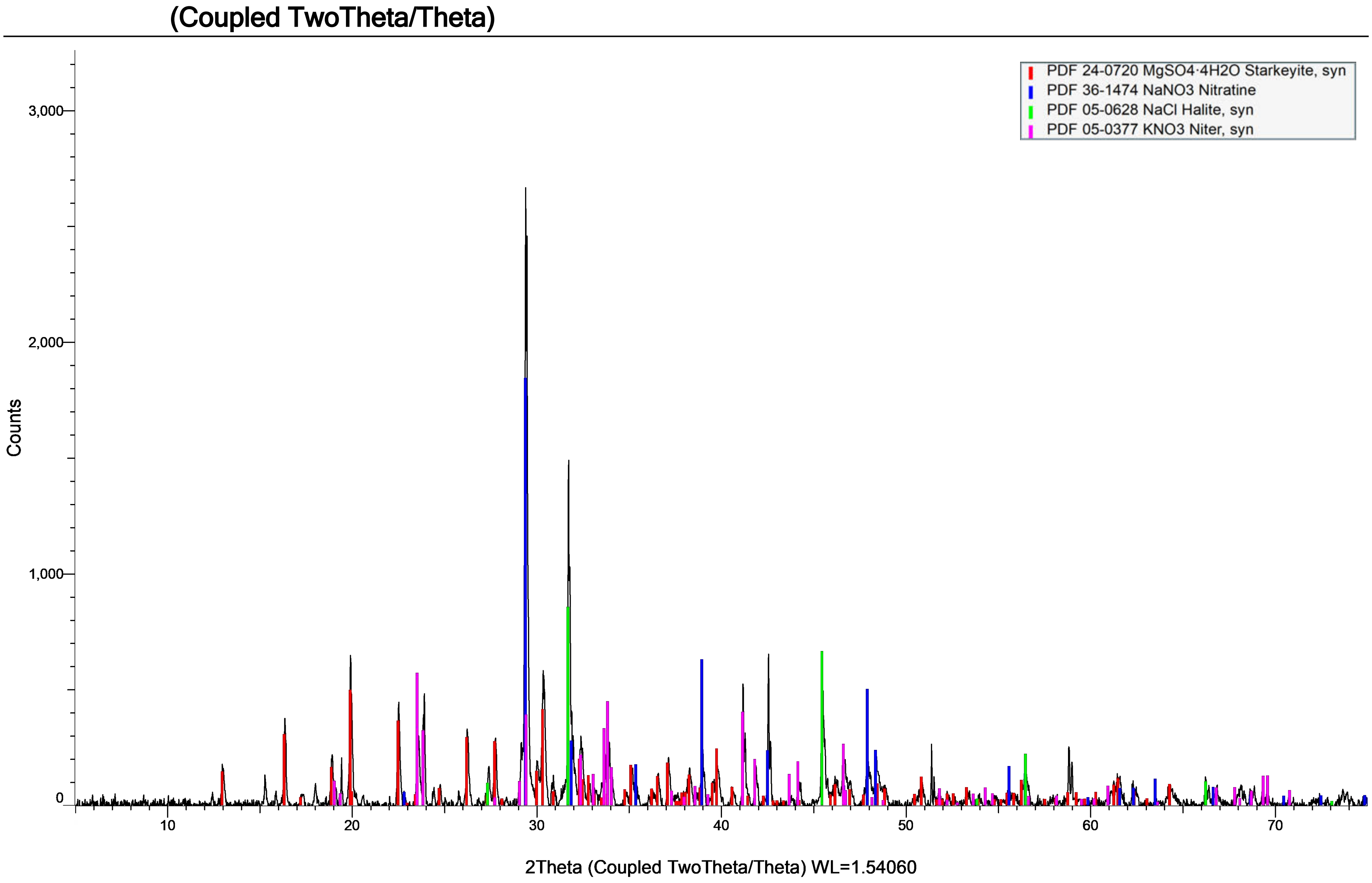

Supplement: Supplementary file 1 — ao4c01486_si_001.pdf [file ao4c01486_si_001.pdf]
